# A Low-Cost Device for Measurement of Exhaled Breath for the Detection of Obstructive Lung Disease

**DOI:** 10.3390/bios12060409

**Published:** 2022-06-13

**Authors:** Adil Ahmad Shahzad, Shafaq Mushtaq, Asim Waris, Syed Omer Gilani, Maha Abdallah Alnuwaiser, Mohammed Jameel, Niaz Bahadur Khan

**Affiliations:** 1 National University of Sciences and Technology (NUST), Islamabad, Pakistan; ahmad.adil28@gmail.com (A.A.S.); asim.waris@smme.nust.edu.pk (A.W.); omer@smme.nust.edu.pk (S.O.G.); 2 Accidents and Emergency Department, Pakistan Institute of Medical Sciences, Islamabad 44000, Pakistan; shiffa92@gmail.com; 3 Department of Chemistry, College of Science, Princess Nourah bint Abdulrahman University, P.O. Box 84428, Riyadh 11671, Saudi Arabia; maalnoussier@pnu.edu.sa; 4 Department of Civil Engineering, College of Engineering, King Khalid University, Asir Abha, Saudi Arabia, P.O. Box: 960 - Postal Code: 61421; jamoali@kku.edu.sa

**Keywords:** exhaled ammonia, hydrogen sulfide, acetone and alcohol, obstructive lung disease, biomarkers, pulmonary function test, device for measurement of exhaled breath

## Abstract

Breath sensor technology can be used in medical diagnostics. This study aimed to build a device to measure the level of hydrogen sulfide, ammonia, acetone and alcohol in exhaled breath of patients as well as healthy individuals. The purpose was to determine the efficacy of these gases for detection of obstructive lung disease. This study was conducted on a total of 105 subjects, where 60 subjects were patients of obstructive lung disease and 45 subjects were healthy individuals. Patients were screened by means of the Pulmonary Function Test (PFT) by a pulmonologist. The gases present in the exhaled breath of all subjects were measured. The level of ammonia (32.29 ± 20.83 ppb), (68.83 ± 35.25 ppb), hydrogen sulfide (0.50 ± 0.26 ppm), (62.71 ± 22.20 ppb), and acetone (103.49 ± 35.01 ppb), (0.66 ± 0.31 ppm) in exhaled breath were significantly different (*p* < 0.05) between obstructive lung disease patients and healthy individuals, except alcohol, with a *p*-value greater than 0.05. Positive correlation was found between ammonia w.r.t Forced Expiratory Volume in 1 s (FEV1) (r = 0.74), Forced Vital Capacity (FVC) (r = 0.61) and Forced Expiratory Flow (FEF) (r = 0.63) and hydrogen sulfide w.r.t FEV1 (r = 0.54), FVC (r = 0.41) and FEF (r = 0.37). Whereas, weak correlation was found for acetone and alcohol w.r.t FEV1, FVC and PEF. Therefore, the level of ammonia and hydrogen sulfide are useful breath markers for detection of obstructive lung disease.

## 1. Introduction

Technology plays an active role in developing medical applications, while making use of information for making important decisions [1]. Breath sensor technology can be used as a tool for medical diagnostics [2]. Gases found in the human breath are important biomarkers of health. The major compounds in exhaled breath are oxygen, CO_2_, water vapor, and nitrogen, with 500 different kinds of components. We can detect various diseases of the respiratory system, metabolic system and digestive system that significantly impact breath gases [1]. Breath gases are produced as a result of various metabolic processes that are released into blood. Once the blood reaches the lungs, the gases are passed on to the airways, for exchange of gases through the mouth [3].

Obstructive lung diseases are diseases of the airways and other structures of the lungs that make it difficult to exhale all the air in the lungs. It may be due to damage or narrowing of the airways [4] caused by asthma, Chronic Obstructive Pulmonary Disease (COPD), infection, allergens, air pollution, exercise, cold air and Gastro Esophageal Reflux Disease (GERD) [5]. According to the “global burden of asthma”, around 300 million people are suffering from asthma and its prevalence is increasing 50% per decade [6]. Some risk factors for lung obstructive disease are things in the environment around us, genes, race, gender, job, lung infection, allergies or obesity, and, with, for example, asthma and COPD, some risk factors run in families. Current methods for detection of obstructive lung disease are the Pulmonary Function Test (Spirometry), X-ray, CT scan, Methacholine, FENO and blood or skin allergy tests [5,7]. According to the US Center for Disease Control and Prevention (CDC), people having moderate to severe obstructive lung disease are at higher risk of developing complications from Covid-19 infection [8].

Human breath analysis is a novel, easy, instant and non-invasive method to diagnose lung obstruction [1]. Some possible breath biomarkers for detection of obstructive lung diseases are nitric oxide (NO), hydrogen per oxide (H_2_O_2_)_,_ carbon monoxide (CO), hydrogen sulfide (H_2_S), alcohol (C_2_H_5_OH), acetone(C_2_H_6_CO) and ammonia (NH_3_) [9,10,11,12,13,14,15]. Previous studies have revealed that the breath levels of nitric oxide, hydrogen peroxide, carbon monoxide and hydrogen sulfide are important for diagnosis of lung obstruction. Moreover, nitric oxide in human breath is an approved marker by the Food and Drug Administration (FDA) for clinical monitoring of asthma [9,10,11,16]. A fast increase of temperature in human breath was reported for obstructive lung patients by Paredi et al., in 2002 [17]. Breath level of ammonia, acetone and alcohol can be important for diagnosis of lung obstruction [13,14,15]. However, limited studies have been conducted for determining the efficacy of ammonia, alcohol and acetone for detection of obstructive lung disease.

The obstructive lung disease diagnosis tests are conventionally high cost, invasive, time consuming and hazardous to health, due to exposure to radiation. This study aimed to fabricate a low-cost device by using Arduino UNO and MQ sensors. It also aimed to measure the level of hydrogen sulfide, ammonia, acetone and alcohol in the exhaled breath of obstructive lung patients and healthy individuals. Moreover, this study found correlation between level of breath gases and severity of obstructive lung disease.

## 2. Material and Methods

### 2.1. Study Subjects

This study was conducted on a total of 108 subjects, with 62 subjects being patients of obstructive lung disease and 47 subjects being healthy individuals. Adults were recruited among patients attending the Pulmonology Outpatient’s Department (OPD) of Military Hospital (MH), Rawalpindi, Pakistan. This experimental protocol was approved by the ethical committee of the National University of Sciences and Technology (NUST), Islamabad, Pakistan (NUST/SMME-BME/REC/000512/32612020)”. Subjects were asked to sign the consent form before participating.

### 2.2. Protocol

A Pulmonary Function Test (PFT) was measured for all the individuals by means of a large MR spirometer (Spriolab, Italy). Measured parameters included: FVC, FEV1 and “FEV1/FVC”, along with PEF. A standard protocol was followed to conduct the PFT. Based on the PFT, obstructive lung patients were screened by a healthcare professional. Individuals were divided into two groups of patients and healthy individuals. Patients were further ranked on a scale of mild, moderate, moderate severe, severe and very severe obstruction by the healthcare professional. None of the patients was treated with glucocorticoids. All subjects were asked not to take any food for at least one hour before the test, since that could impact the results. Those who did not follow the protocol were either excluded from the study or were asked to wait for one hour. The level of breath markers was recorded for patients and healthy individuals one by one. All individuals were asked to take a deep inhale breath and strongly blow on to the sensors placed in front of them for at least 4 s (in one cycle of breathing). Individuals who could not blow on the sensors by protocol were asked to blow on the sensors again, to make the breath recording process uniform.

**Figure 1:**
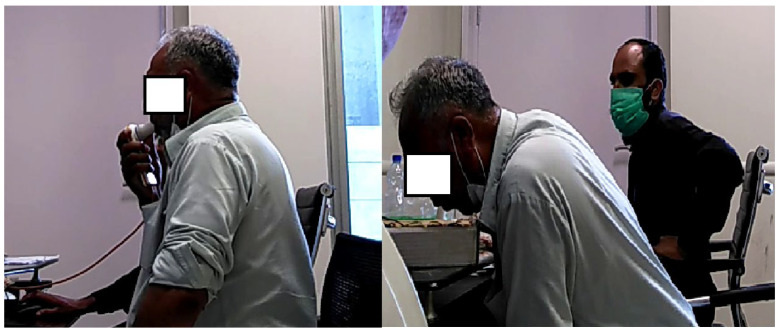
Recording of Pulmonary Function Test (PFT) on the left and recording of Exhaled Breath Compounds on the right.

Figure 1 shows the recording of the Pulmonary Function Test and recording of exhaled breath compounds.

### 2.3. Health Status

Clinical data was collected for all the individuals comprising the enrolment: gender, age, weight, smoking history and medical history. Furthermore, a spirometry test was conducted to confirm the health status of the patients.

### 2.4. Experimental Setup

#### 2.4.1. Device Components

A device was fabricated to measure the level of concentration of various gases (ammonia, hydrogen sulfide, acetone, alcohol) in exhaled breath. This device consists of a circuit of four Mingan-Qi-lai (MQ) sensors, Arduino UNO, a load resistor and a digital temperature humidity sensor (DHT22). The MQ sensors were used to find the concentration levels of four different gases. A digital temperature humidity sensor was connected with the Arduino UNO to feed digital signals of humidity and temperature to Arduino UNO. Arduino UNO was used to get analog and digital values from the sensors, to compute corresponding results by performing mathematical calculations and to display these results on a laptop through a serial monitor. The load resistor was connected to adjust sensor sensitivity and accuracy.

#### 2.4.2. Working of MQ Sensor

The MQ sensor series contains tin oxide (SnO_2_)-based electrochemical gas sensors. It computes concentration of the specific gas using a voltage divider network present in the sensor. It also contains a small heater fabricated along with the sensor to help in measuring different gases. Electrical resistance of tin oxide changes according to concentration of the measured gas. These sensors work on a 5V DC voltage; therefore, the corresponding analog value of the sensor is achieved from a voltage divider circuit [18].

#### 2.4.3. Interfacing Components

**Figure 2:**
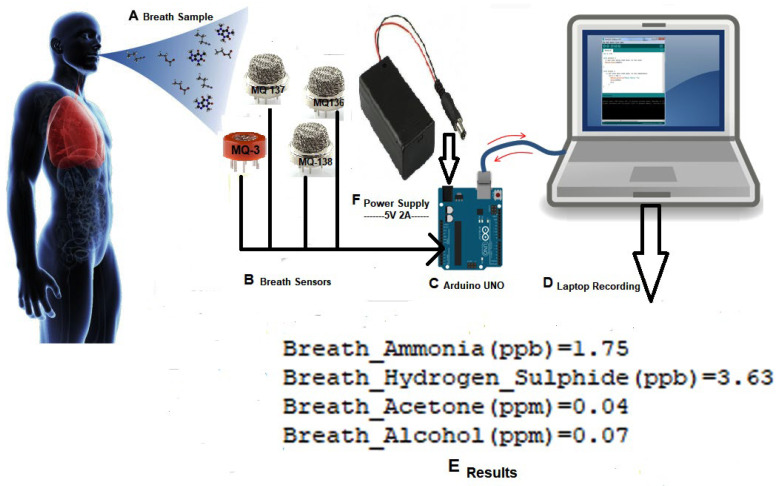
Diagram of Device Design and Interfacing.

Gas sensors were placed on the Vero-board and Breadboard. All connections were made with the Arduino UNO. Power and ground pins of the gas sensors were attached to +5 V and ground terminals of the Arduino, respectively. Arduino UNO was directly connected with the laptop, through which we could control and access data. Figure 2 displays the device design of the system.

### 2.5. Data Analysis Statistics

All datasets were stored in a spread sheet. The normality of all the datasets was tested by the Kolmogorov-Smirnov (S-R) test. Normally distributed data was represented by mean and standard deviation, while non-normally distributed data was represented by median and standard deviation. For correlation analysis, datasets of both groups were compared using analysis of variance (ANOVA-I), tables and bar plots. Pearson linear regression analysis was done between level of breath gases and predicted % of Pulmonary Function Test variables. Conclusions were drawn from the results of the statistical analysis.

## 3. Results

A total of 105 individuals (87 males and 18 females) enrolled. All the hypotheses were tested by analysis of variance for 60 patients and 45 healthy individuals, and used breath hydrogen sulfide, ammonia, acetone and alcohol as independent variables and obstructive lung disease as dependent variable.

### 3.1. Statistical Comparison Analysis

#### 3.1.1. Exhaled Hydrogen Sulfide

Statistical analysis of hydrogen sulfide in breath of obstructive lung patients (32.29 ± 20.83 ppb) and healthy subjects (62.71 ± 21.20 ppb) showed significant difference (*p* < 0.0001).

#### 3.1.2. Exhaled Alcohol

Statistical analysis of alcohol in breath of obstructive lung patients (0.96 ± 0.52 ppm) and healthy subjects (1.09 ± 0.51 ppm) showed non-significant difference (*p* > 0.05).

#### 3.1.3. Exhaled Ammonia

Statistical analysis of ammonia in breath of obstructive lung patients (68.83 ± 35.25 ppb) and healthy people (103.49 ± 35.01 ppb) showed significant difference (*p* < 0.0001).

#### 3.1.4. Exhaled Acetone

Statistical analysis of acetone in breath of obstructive lung patients (0.50 ± 0.26 ppm) and healthy people (0.66 ± 0.31 ppm) showed significant difference (*p* < 0.001).

Table 1 displays analysis of variance of all independent variables for patients and healthy individuals. Significant difference was observed for all the gases in exhaled breath of patients and healthy individuals, except alcohol.

**Figure 3:**
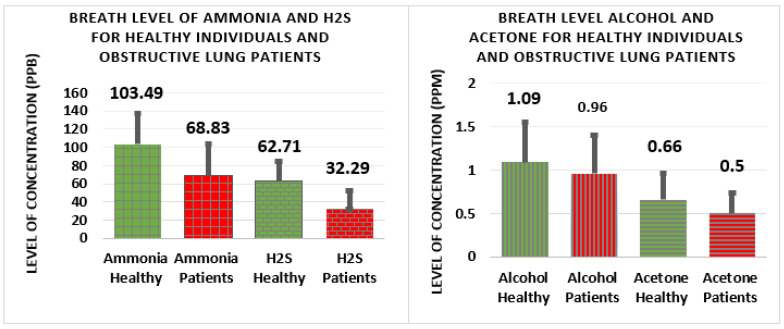
Displays the mean and standard deviation level of exhaled breath ammonia and hydrogen sulfide on the left and exhaled breath alcohol and acetone on the right between patients and healthy individuals.

In Figure 3, the bar plots display the mean and standard deviation level of alcohol and acetone on the left, and of ammonia and hydrogen sulfide on the right, for obstructive lung patients and healthy people.

Table 2 displays the general characteristics of subjects: number, gender, age, weight, smoking status and clinical characteristics. It also displays severity of disease, level of ammonia, hydrogen sulfide, acetone and alcohol in exhaled breath. FVC, FEV1 and FEV1/FVC are highlighted for all subjects. Moreover, it shows the comparison analysis that, for all the independent variables, a significant difference of level was found between patients and healthy individuals. Moreover, the breath levels of ammonia and hydrogen sulfide decreased with severity of obstructive lung disease. Lower levels of breath ammonia, hydrogen sulfide, acetone and alcohol were observed for patients with severe and very severe obstruction.

## 4. Discussion

In this study analysis of several gases in exhaled breath of obstructive lung patients and healthy individuals was performed. Our study demonstrates that breath level of hydrogen sulfide, alcohol, acetone and ammonia found in a healthy individual is different than that found in patients of lung obstruction. Similarly, PFT parameters (FVC, FEV1, PEF) found in healthy individuals are different than those in patients of obstructive lung disease.

If certain foods are taken before the test, they affect the concentration level of measured gases, resulting in false positive or false negative results. Therefore, all subjects were instructed to avoid taking any food for at least one hour before the test. This was the limitation of the exhaled breath recording test.

### Statistical Correlation Analysis

**Figure 4:**
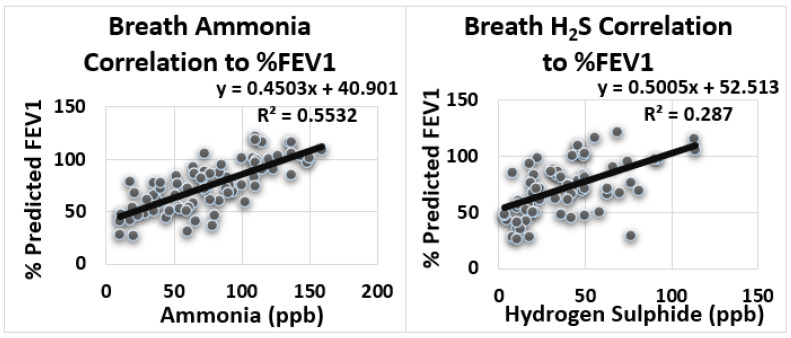
Correlation between exhaled breath ammonia and hydrogen sulfide to Forced Expiratory Volume in 1 s (FEV1) (% of predicted).

Figure 4 displays the large positive correlation between level of ammonia and FEV1 (r = 0.74) (R^2^ = 0.553). A large positive correlation was detected between level of hydrogen sulfide and FEV1 (r = 0.54) (R^2^ = 0.287).

**Figure 5:**
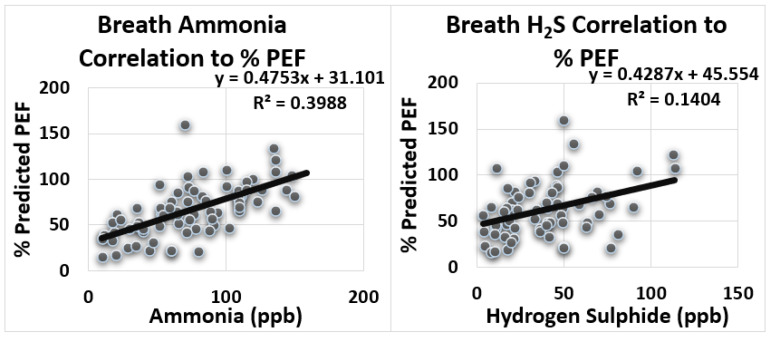
Correlation between exhaled breath ammonia and hydrogen sulfide to peak expiratory flow (PEF) (% of predicted).

Figure 5 displays the positive correlation between level of ammonia and PEF (r = 0.38) (R^2^ = 0.398). A medium positive correlation was perceived between level of hydrogen sulfide and PEF (r = 0.38) (R^2^ = 0.14).

Table 3 displays the summary of Pearson linear regression analysis, which was performed between exhaled breath variables and PFT variables. Medium correlation was observed between exhaled breath ammonia and FEV1, FVC and PEF. Small correlation was observed between hydrogen sulfide and FEV1, FVC and PEF. No significant correlation was observed for acetone and alcohol with FEV1, FVC and PEF.

This clinical study found correlation between the studied exhaled gases of obstructive lung disease patients. It also found reference values for those breath markers that are rarely studied for lung obstruction. These findings are important for use of breath gases, other than nitric oxide, for the monitoring of obstructive lung disease. As lung obstructive disease causes narrowing of airways, the levels of gases in the exhaled breath of obstructive lung patients is lower.

Like nitric oxide and carbon monoxide, hydrogen sulfide is called the third gasotransmitter. Hydrogen sulfide is produced as a result of the relaxing action of vascular smooth muscles [19]. Zhang et al. established, in 2014, that the breath level of hydrogen sulfide is positively correlated with the percentage of predicted forced expiratory volume (FEV) and Asthma Control Test (ACT) score [20]. Similarly, our study demonstrates that the breath level of hydrogen sulfide in healthy individuals (63.98 ± 21.63 ppb) is significantly higher than the breath level of hydrogen sulfide in obstructive lung patients (34.12 ± 20.17 ppb). Moreover, a positive correlation was observed for ammonia and hydrogen sulfide with FEV1, FVC and PEF. Kinoyama et al. established, in 2008, that the average value of acetone in healthy individuals is 0.53 ± 0.45 ppm [21]. Our study highlights that the level of acetone in the breath of healthy individuals (0.66 ± 0.31 ppm) is significantly more than the level of acetone in the breath of patients with obstructive lung disease (0.50 ± 0.26 ppm); *p* < 0.05. Carraro et al., in 2005, measured condensed ammonia from the saliva of asthmatic children. Ammonia condensate for asthmatic children (Treated: 476.17 μM, Non-treated: 253.24 μM) is lower as compared to healthy children (Healthy: 788.3 μM) [13]. Similarly, our study identified a significant decrease in breath ammonia for obstructive lung patients (68.83 ± 35.25 ppb) in comparison to that of healthy individuals (103.49 ± 35.01 ppb) (*p* < 0.05). Most research conducted on breath ammonia has been related to the detection of kidney disease, as it is an accepted breath marker for the monitoring of kidney disease [22]. Yates et al., in 1996, measured the effect of alcohol ingestion on the level of exhaled nitric oxide. They found a significant decrement in the level of breath nitric oxide occurred in asthmatic patients (204 ± 58 ppb) without ingestion of alcohol (from 204 ± 58 ppb to 158 ± 59 ppb), while no significant decrement occurred in healthy individuals (from 122 ± 14 ppb to 114 ± 15 ppb) [14]. Our study demonstrated no significant difference found in the level of breath alcohol for obstructive lung patients (0.96 ± 0.25) and healthy people (1.09 ± 0.51) (*p* > 0.05). The Cyranose 320 electronic nose device consists of 32 composite sensors that can be used to measure exhaled breath [23]. Nidheesh et al., in 2022, used the Cyranose 320 electronic nose device to measure the exhaled breath of post covid-19 asthma subjects. They found a significant difference in values of sensors 5, 23 and 21. These sensors gave noticeable responses to aldehydes, ketones, amines and alcohol [24].

## 5. Conclusions

The levels of ammonia and hydrogen sulfide are useful breath biomarkers of obstructive lung disease because large and medium positive correlations were found for ammonia and hydrogen sulfide to variables of the Pulmonary Function Test. Although significant difference was found for the level of acetone between patients and healthy subjects, the levels of acetone and alcohol were not correlated with variables of the Pulmonary Function Test. Therefore, breath concentration of acetone and alcohol are not biomarkers of obstructive lung disease and the level of ammonia and hydrogen sulfide may be used to detect obstructive lung disease. Further studies are required to compare the performance of exhaled breath ammonia and hydrogen sulfide to FeNO for the monitoring of obstructive lung disease.

## Figures and Tables

**Table 1:** Comparison analysis of gases in exhaled breath between obstructive lung disease patients and healthy subjects.

S.N	Dependent Variable	Independent Variables (Exhale Breath Compounds)	*p*-Value	Conclusion
1	Obstructive Lung Disease	Alcohol (C_2_H_5_OH)	*p* > 0.05	Insignificant difference
2		Acetone (CH_3_COCH_3_)	*p* < 0.001	Significant Difference
4		Ammonia (NH_3)_	*p* < 0.0001	Significant Difference
5		Hydrogen Sulfide (H_2_S)	*p* < 0.0001	Significant Difference

**Table 2:** Comparison of demographic and clinical characteristics. Significant difference of clinical characteristics was found for obstructive lung patients and healthy subjects.

Information Group	Patients	Healthy
Number	60	45
Gender (M/F)	45/15	42/3
Average Age (Years)	45.66 ± 15.08	42.22 ± 14.84
Average Weight (Kg)	67.76 ± 11.51	70.5 ± 12.41
Smoking Status(Smoker/Non-smoker)	12/48	11/34
Overall Average level of exhaled gases	NH_3_ (68.83 ± 35.25ppb), H_2_S (32.29 ± 20.83 ppb), C_2_H_6_CO (0.50 ± 0.26 ppm), C_2_H_5_OH (0.96 ± 0.52 ppm)	NH_3_ (103.49 ± 35.01 ppb), H_2_S (62.71 ± 22.20 ppb), C_2_H_6_CO (0.66 ± 0.31 ppm), C_2_H_5_OH (1.09 ± 0.51 ppm)
Severity of Lung Disease and Average plus STD level of exhaled Gases		
Mild Obstruction (number, level of exhaled gases)	21, NH_3_ (88.11 ± 32.66 ppb), H_2_S (37 ± 18.33 ppb), C_2_H_6_CO (0.51 ± 0.23 ppm), C_2_H_5_OH (0.92 ± 0.17 ppm)	same as above
Moderate Obstruction (number, level of exhaled gases)	10, NH_3_ (65.77 ± 32.07 ppb), H_2_S (42.36 ± 22.56 ppb), C2H6CO (0.56 ± 0.25 ppm), C2H5OH (1.07 ± 0.57 ppm),	same as above
Moderate Severe Obstruction (number, level of exhaled gases)	10, NH_3_ (55.18 ± 22.86 ppb), H_2_S (23.24 ± 15.72 ppb), C_2_H_6_CO (0.39 ± 0.28 ppm), C_2_H_5_OH (0.84 ± 0.63 ppm)	same as above
Severe Obstruction (number, level 0f exhaled gases)	14, NH_3_ (58.14 ± 38.66 ppb) H_2_S (26.31 ± 20.39 ppb) C_2_H_6_ CO (0.48 ± 0.28 ppm) C2H5OH (0.0.93 ± 0.42 ppm)	same as above
Very Severe Obstruction (number, level of exhaled gases)	5, NH_3_ (43.32 ± 21.35 ppb) H_2_S (21.36 ± 19.52 ppb) C_2_H_6_CO (0.404 ± 0.34 ppm) C_2_H_5_OH (1.41 ± 0.51 ppm)	same as above
Average plus STD level of % of Predicted FVC	76.93 ± 23.08	100.27 ± 10.11
Average plus STD level of % of Predicted FEV1	60.60 ± 18.59	98.91 ± 12.40
Average plus STD level of % of Predicted FVC/FEV1	81.18 ± 17.31	101.82 ± 9.19
Average level of % of Predicted PEF	49.4 ± 19.88	88.14 ± 20.14

**Table 3:** Pearson Linear Regression Analysis of Exhaled Breath variables and Lung Function Test Variables.

S.N	Variables	Equation	R (Correlation Coefficient)	R^2^ (Determination Coefficient)	Summary
1	NH_3_-FEV1	y = 0.45x + 40.90	0.74	0.553	Large Positive Correlation
2	H_2_S-FEV1	y = 0.50x + 52.51	0.54	0.287	Medium Positive Correlation
3	NH_3_-FVC	y = 0.34x + 61.27	0.61	0.377	Medium Positive Correlation
4	H_2_S-FVC	y = 0.36x + 69.67	0.41	0.172	Small Positive Correlation
5	NH_3_-PEF	y = 0.47x + 31.10	0.63	0.398	Medium Positive Correlation
6	H_2_S-PEF	y = 0.429x + 45.55	0.37	0.14	Small Positive Correlation

## Data Availability

All data generated or analysed during this study are included in this published article.
